# Impact of pandemic on mental health in lower- and middle-income countries (LMICs)

**DOI:** 10.1017/gmh.2020.28

**Published:** 2020-12-03

**Authors:** Manasi Kumar, Pushpam Kumar

**Affiliations:** 1 Department of Psychiatry, University of Nairobi College of Health Sciences, University of Nairobi, Nairobi 00100, Kenya; 2 UN Environment Programme (UNEP), P.O. Box 30522, Nairobi 0010, Kenya

## Introduction

Globally, the COVID-19 pandemic has aggravated poverty and resulted in mental illnesses. This nexus of rising mental illness in low resource contexts and increasing extreme poverty in lower- and middle-income countries (LMICs) in the context of the global pandemic is now blatantly visible. These trends have not only exacerbated extreme poverty, but have also induced psychological distress and ill-health, thus significantly affecting general mental health functioning of people across the globe. We ask for urgent action in developing policy that addresses poor mental health, which emanates from material deprivation and is currently intensified by the prevailing pandemic. Our commentary highlights linkages between incidence of poverty and the rise of mental illnesses globally in the context of the COVID-19 pandemic.

Mental ill-health and poverty are closely linked in a complex negative cycle. It has been found that except for diseases such as schizophrenia, where the genetic factor could be a dominant factor, extreme material deprivation resulting from the lack of gainful employment, inadequate nutrition, and lack of basic amenities could precede mental illness (Patel *et al*., 2018). People affected by material deprivation are known to be at a higher risk of mental illness (World Health Organization, 2013).

## Poverty and mental health

Extreme poverty, measured as earning less than USD 1.90 a day by the World Bank, is accompanied by hunger, overcrowded living conditions, low accessibility and affordability of health services, limited educational and livelihood opportunities, and adverse life events associated with socioeconomic vulnerabilities. The culmination of these factors is known to increase risk of mental distress and mental illnesses (Lund *et al*., 2011). Over time an awareness in the field of mental health recognizes that this vicious cycle of poverty and mental ill health needs to be viewed from human rights perspective. In addition, the focus on poverty alleviation within health sciences needs a better alignment with the global development agenda (Patel *et al*., 2018; Kumar, 2019). In general, people living in poverty also experience a lower quality of life and exhibit low well-being indicators, such as poor health, social and physical security, and political freedom and choices. Poverty increases the risk of mental illnesses that consequentially increase the likelihood of economic downturn at individual and family levels (World Health Organization, 2011). Individuals with mental illness are stigmatized, actively discriminated against, and their ability to hold livelihoods and access opportunities are reduced. The cost associated with mental health treatments also increases economic burden on the individual and the family (Patel *et al*., 2018). This indicates that living with mental illness over time can also make one poorer.

Before COVID-19, one out of four people suffered from mental ill-health (primarily anxiety and depression). Now as approximately 3–4 billion people are placed in lockdown as a mitigation response measure to COVID-19, more and more people are suffering from anxiety. People are under acute stress stemming from job loss, COVID-19-related deaths (within family and friends), and abnormal situations associated with restriction of movement and social isolation. In addition, a large number of individuals experience stress and anxiety from suspected exposure to COVID-19 and from being quarantined away from the secure confines of their homes in LMICs.

## Rise in mental health needs during COVID-19 pandemic

COVID-19 is merely one symptom of the dysfunctional relationship between humans and nature, which is manifested through climate change, loss of biodiversity, habitat fragmentation, and domestication of wild animals (UNEP, 2020). In 2016, the United Nations Environment Programme flagged the worldwide increase in zoonotic epidemics. Around 60% of all infectious diseases in humans are zoonotic, and the emergence of zoonotic diseases is closely linked with the depleting health of ecosystems (UNEP, 2016).

There is emerging evidence from studies in Wuhan, China, where the first lockdown was initiated as a measure to prepare for effective responses to COVID-19, that indicates that the lockdown in itself has severely impacted the mental health of the Chinese people. Fu *et al*. (2020) report in a nationwide cross-sectional study that easing of the lockdown in the city was explicitly associated with stressful impact on a sizeable number of participants (Ma *et al*., 2020). Measures such as quarantine, isolation, and social distancing have seriously impacted the mental health and general life of the people in China. Experts have arrived at the consensus that these measures have triggered a wide variety of psychological disorders, such as panic disorder, anxiety, and depression in the general population (Qiu *et al*., 2020).

The results of a Chinese study reviewing the effect of COVID-19 mitigation plans on mental health led Wuhan's Government officials to develop a multi-layered response that involved forming psychological intervention teams for key populations that are likely to be impacted by exposure to COVID-19 and its adverse risk factors (Kang *et al*., 2020). These initial studies from China have highlighted the need to address vulnerable populations, improve psychosocial and medical care access, ramp up disaster preparedness and mitigation, and strengthen health systems, disaster management and intervention (Qiu *et al*., 2020).

Public health emergencies impact both individuals and communities. There are short- and long-term consequences for mental health and wellbeing, as witnessed in the post-epidemic phases of Ebola, SARS, and MERS (Galea *et al*., 2020). During epidemics, in addition to a large number of the population suffering from depression and anxiety, surges in maladaptive high-risk behaviors also increased, such as domestic violence and interpersonal conflicts. Issues surrounding fear, panic, and discrimination are being reported (Usher *et al*., 2020) and are covered daily in news and social media. Opportunities to monitor psychosocial needs and deliver intervention support during direct patient encounters in clinical practice are curtailed in this crisis by large-scale home confinement (Pfefferbaum and North, 2020). It is estimated that the prevalence of posttraumatic stress disorder in the general population ranges from 4% to 41% and the prevalence of major depression will increase by 7% after the outbreak (Torales *et al*., 2020).

The pandemic may worsen existing mental health problems and lead to more cases among children and adolescents. This is due to the unique combination of the public health crisis, social isolation, disruption of education, limited peer interactions, boredom, and economic recession. All of these factors negatively impact wellbeing (Golberstein *et al*., 2020). For those with existing and underlying health conditions, these worries are further exacerbated due to the mass quarantine (Rubin and Wessely, 2020). Given that globally 36 million people have been infected, out of which approximately more than 1 million deaths have been recorded as of 7 October 2020 (World Health Organization, 2020a), we know that the mental health needs of individuals who are infected and those who have recently recovered may be significantly high. The isolation and loneliness around palliative care of those who succumb to the illness also means that the families of these individuals experience painful bereavement and traumatic grief. In many parts of the world, relatives and family members are stigmatized and forced into quarantine. Such measures are on the verge of becoming human rights violations (World Health Organization, 2020b) and efforts are needed to uphold rights and civil liberties.

## Pandemic aggravating poverty in under-resourced settings

Consequences of COVID-19 lockdown and mitigation efforts have exacerbated the existing incidence of poverty and inequality among and within countries. Recently, the World Bank projected that roughly 71 million of the world's population will be pushed into extreme poverty, assuming that the COVID-19 pandemic does not change inequality within countries or that the nation's growth accumulates equally to everyone. Sub-Saharan Africa and South Asia are anticipated to be the hardest hit regions, with 26 million and 32 million of the people in each region, respectively, projected to be living on USD 1.90 or less a day (Gerzon *et al*., 2020).

Alongside monetary poverty, there is also acute multidimensional poverty, in which households are deprived of one-third or more of the 10 indicators of health, education, and living standards set by the Sustainable Development Goals. The global Multidimensional Poverty Index estimates that the 2% (or 10 million) of the world's population, out of the 39 million poor people, experience severe acute multidimensional poverty and another 7.7% (or 40 million people) have not reached, but are vulnerable to acute multidimensional poverty (UNDP, 2020). The indicators of multidimensional poverty are when a household has a malnourished member, high child mortality, none in the family has completed 6 years of education, children do not attend school, clean drinking water is lacking, nourishing food is lacking, improved sanitation is lacking, and adequate safe housing is also lacking. People experiencing acute multidimensional poverty are at extremely high-risk for COVID-19 and are often unable to comply with the preventative measures of social isolation (UNDP, 2020). This estimated projection of increase in monetary global poverty (USD 5.5/day) means that any progress in eliminating extreme poverty would be set back by at least 20 years (World Bank, 2020a, b).

The impact of COVID-19 on the incidence of poverty is obvious and encompassing. The world is experiencing a decline in gross domestic product between 4% and 8% in 2020 (International Monetary Fund, 2020). The restriction on movement of people, logistic bottlenecks emanating from the lockdown, and in large cases, the closure of production units as a safety and health response has severely compromised the ability of the people to supply labor and earn wages. The impact is apparent in the extreme and marginal poor (less than USD 1.9 a day) living in LMICs. For example, due to COVID-19, sub-Saharan Africa would see a rise in people living under extreme poverty to 50 million (42%) by 2021 against the pre-COVID-19 estimates of 37.8% (World Bank, 2020a, b).

In general in LMICs, the hard-hit sectors have a high proportion of informal workers and with limited access to health services and social protection. Without appropriate policy measures, such workers risk falling into extreme poverty and will experience challenges in regaining livelihoods during the recovery period.

In a recent COVID-19 mental health impact survey conducted by the WHO, results indicate the pandemic has disrupted or halted critical mental health services in 93% of countries worldwide while the demand for mental health is increasing (World Health Organization, 2020c). We know that COVID-19 is straining the capacity of public health and essential services across the world. There are worries that the LMICs will be severely affected because investments in adequate staffing, health equipment, access to health services, and evidence-based guidelines and their timely dissemination and coordinated implementation continues to be a challenge. Non-COVID-19 patients would suffer as much as those in need of serious medical intervention. Recommended interventions for COVID-19 involve (1) the assessment of the accuracy of information, (2) enhancing social support, (3) reducing the stigma associated with the disease, (4) maintaining a normal life while adhering to safety measures, and (5) using psychosocial services, particularly online services, when needed (Rajkumar, 2020). Most of these responses are compromised in under-resourced settings where health systems cannot cope with preventive and promotive messaging and support. The treatment burden is in itself so significant that prevention and ancillary support services cannot be rallied in a timely manner.

Those most affected in this pandemic are vulnerable low wage earning individuals who also have underlying chronic conditions. This is worsened if they also belong to vulnerable groups, such as elderly, migrant workers, marginalized ethnic or racial groups, women, pregnant women, and individuals with mental illnesses. In such cases, access to services and available support is also compromised (Pfefferbaum and North, 2020; Rajkumar, 2020). Forced quarantine and marking of informal settlements and localities as ‘red zones’ due to COVID-19 exposure borders human and mental health rights violation. Isolating people without adequate provisioning of their everyday life needs can aggravate people's stress and helplessness (Rubin and Wessely, 2020; Torales *et al*., 2020). Infectious diseases bring with them heightened fear of contagion, stigma, and social ostracization of individuals and communities, as was seen during HIV, Ebola, and related epidemics.

There is existing evidence that poor mental health can be a cause and a consequence of poverty and destitution (Lund *et al*., 2011; Funk *et al*., 2012). Common mental health disorders affect the wage-earning capacity of the poor as the individual's everyday functioning is reduced (Kleinman, 2009). Challenging mental health condition also reduces the wage earning potential of individual because of lower employability. The absolute material deprivation can also cause depression, anxiety, or traumatic stress. We know that absolute poverty in many parts of the world can be intergenerational. Pandemics further hinder the capability of the poor to climb out of extreme poverty. Even though mental health disorders affect 1 billion people globally, the governments of the world spend only about 2% of their health budgets on addressing this issue. Additionally, most people lack access to basic psychosocial and psychiatric treatments. The interconnections between poverty and mental health stop short of examining the health and care infrastructure and ignore the political factors that perpetuate an increase in both (Patterson *et al*., 2020).

## COVID-19: driver of poverty and deprivation

The International Monetary Fund suggests that the world's economy is projected to contract by up to 8% in 2020, which is worse than the 2008–09 financial crisis (IMF, 2020). The emerging markets and developing economies are projected to contract by 1% in 2020. On employment, the International Labour Organization forecasts that around 2.7 billion workers are affected due to lockdown (81% of the world's workforce) (International Labour Organization, 2020). By now, several global agencies and think tanks have conclusively shown that workers who are either extremely poor (less than USD 1.90 a day) or poor would be hit the hardest. This includes refugees, migrant workers, non-farm laborers, and workforce engaged through other informal activities.

Although governments have responded promptly with relief packages to keep their economies on life support and to maintain economic activities, informal workers (about 2 billion) are not benefiting from these relief packages. Informal labor has 92.70% of total labor share in low-income countries, 83% in lower-middle countries, 55.35% upper-middle countries, and 14.43% in high-income countries. The University of Oxford's COVID-19 Government Response Stringency Index places India, Pakistan, Nigeria, and Brazil at high risk of their people falling into poverty due to their higher share of the workforce operating within the informal economy (Hale *et al*., 2020).

Poverty affects children disproportionately. One out of five children lives in extreme poverty and ensuring social protection for children and other vulnerable groups is critical. Progress in reducing working poverty has slowed over the past 5 years and efforts need to be reinvigorated (United Nations, 2019). The situation remains particularly alarming in sub-Saharan Africa, where the proportion of working poor has reached 38% in 2018. In the least developed and landlocked developing countries, at least one-quarter of workers live in extreme poverty despite having a job. Employed young people (between 15 and 24 years of age) are more likely to be living in poverty, with a working poverty rate that is double than that of adult workers. The world is already struggling to achieve the target for poverty alleviation set under the globally agreed agenda of the Sustainable Development Goals.

Conclusively, elements of mental health conditions determine the material conditions of the affected individuals who will be unable to sustain life and livelihoods. In the context of a global pandemic, prevailing institutions and governance structures have proved to be a hindrance to the poor in the attainment of minimum material condition, thus making them increasingly vulnerable, marginalized, and sometimes even excluded from social protective nets. The condition of extreme deprivation makes marginalized and weak populations psychologically fragile and vulnerable. A large number of poor populations in sub-Saharan Africa and South Asia have co-morbid health conditions, such as HIV, tuberculosis, cancer, diabetes, and cardiovascular disease, all of which have severe impacts on emotional and physical capability that leads to a compromised psychological functioning.

Our commentary underscores how the world's materially deprived are severely impacted due to this pandemic and the impact will likely continue beyond 2020. The impact of COVID-19 on mental health will also hinge on the economic downturn. Public mental health interventions should be formally integrated into public health preparedness and emergency response plans so that the poor and vulnerable populations can be protected from spiraling into an abject material condition. To break this nexus, a concerted effort to tackle mental health as a sizeable portion of overall health response is the need of the hour. The UN Secretary-General perceptively reiterates the slogan ‘no health without mental health’, which should be the core mandate of global development agenda (TIME, 2020). The mental health impacts associated with COVID-19 need to be assessed, quantified, and a proper response strategy needs to be designed in a nuanced and structured manner. The issue of mental health in poorer parts of the world is not well understood, and typically the remedies are also stigmatized and interventions inadequately resourced.
